# Using Drugs to Slow Aging in the Nematode Caenorhabditis Elegans

**DOI:** 10.1093/geroni/igaf122.2054

**Published:** 2025-12-31

**Authors:** David Gems, Aihan Zhang

**Affiliations:** University College London, London, United Kingdom; University College London, London, United Kingdom

## Abstract

With its 2-3 week lifespan, C. elegans is a convenient animal model for testing drug effects on aging. We have used liposome-mediated drug delivery to reduce confounding effects of interactions between drugs and the nematodes’ bacterial food source, improve compound uptake, and greatly reduce drug quantities needed for trials. We tested six compounds previously reported to extend lifespan: vitamin C, N-acetylcysteine, glutathione (GSH), trimethadione, thioflavin T (ThT), and rapamycin. Life extension was reproduced for the latter four. However, for GSH and ThT, antibiotics abrogated life extension, implying a bacteria-mediated effect; in the latter case, life extension was due to an antibiotic effect of ThT, which protected elderly nematodes against life-limiting infection. For rapamycin, treatment throughout adulthood had a dose-dependent effect, causing a maximal 21.9% increase in mean lifespan, but shortening of lifespan at the highest dose, suggesting drug toxicity. Notably, treatment initiated late in life, from day 16 (or ∼70 years in human terms), robustly increased lifespan. The rapalog temsirolimus extended lifespan similarly to rapamycin. As in mouse, rapamycin had mixed effects on age-related pathologies, inhibiting one (uterine tumor growth) but not several others, suggesting a segmental antigeroid effect.

